# Prize Essay

**Published:** 1841-06

**Authors:** 


PRIZE ESSAY.

The newspapers of Nashville announce that Professor Yandell, of the Louisville Medical Institute, obtained the Premium, offered by the Medical Society of Tennessee a year since, for the best dissertation on Bilious Fever.

